# Clinical Correlation of Cerebrospinal Fluid Total tau Levels and MMSE Score in a memory clinic

**DOI:** 10.1093/geroni/igaa057.3240

**Published:** 2020-12-16

**Authors:** Neil Tangal, Neeraj Tangal, Anil Nair, Malini Nair

**Affiliations:** Alzheimer’s Disease Center, Quincy, Massachusetts, United States

## Abstract

Tau protein levels in cerebrospinal fluid are a biomarker of Alzheimer’s disease. We correlated MMSE severity to CSF tau levels in a large memory clinic sample. We retrospectively analyzed data from patients attending a memory clinic in the south shore of Boston from 2010 to 2020, and had a lumbar puncture to obtain CSF Tau levels. We compiled cognitive screen data from MMSE scores. Univariate analyses used Spearman correlation as data were non-normal. A multivariate model was created including covariates of age, sex, and race. 965 patients attended the memory clinic from 2010 to 2020. 711 had available MMSE scores. 129 subjects had lumbar punctures and available CSF tau levels. Univariate analyses showed that cognition as measured by MMSE total was not correlated to total tau levels in the CSF (rho=-0.07, p>0.05), but caucasian race was inversely associated with CSF tau levels (rho= -0.217, p<0.05). In a multivariate model, tau levels in the CSF were not associated with MMSE, race, gender, or age. In a large memory clinic sample, CSF tau levels did not correlate to MMSE scores, age, race or gender.

